# Recognition of early stage thigmotaxis in Morris water maze test with convolutional neural network

**DOI:** 10.1371/journal.pone.0197003

**Published:** 2018-05-03

**Authors:** Akinori Higaki, Masaki Mogi, Jun Iwanami, Li-Juan Min, Hui-Yu Bai, Bao-Shuai Shan, Harumi Kan-no, Shuntaro Ikeda, Jitsuo Higaki, Masatsugu Horiuchi

**Affiliations:** 1 Department of Molecular Cardiovascular Biology and Pharmacology, Ehime University, Graduate School of Medicine, Toon, Ehime, Japan; 2 Department of Cardiology, Pulmonology, Hypertension and Nephrology, Ehime University, Graduate School of Medicine, Toon, Ehime, Japan; 3 Department of Pharmacology, Ehime University, Graduate School of Medicine, Toon, Ehime, Japan; Tokai University, JAPAN

## Abstract

The Morris water maze test (MWM) is a useful tool to evaluate rodents’ spatial learning and memory, but the outcome is susceptible to various experimental conditions. Thigmotaxis is a commonly observed behavioral pattern which is thought to be related to anxiety or fear. This behavior is associated with prolonged escape latency, but the impact of its frequency in the early stage on the final outcome is not clearly understood. We analyzed swim path trajectories in male C57BL/6 mice with or without bilateral common carotid artery stenosis (BCAS) treatment. There was no significant difference in the frequencies of particular types of trajectories according to ischemic brain surgery. The mouse groups with thigmotaxis showed significantly prolonged escape latency and lower cognitive score on day 5 compared to those without thigmotaxis. As the next step, we made a convolutional neural network (CNN) model to recognize the swim path trajectories. Our model could distinguish thigmotaxis from other trajectories with 96% accuracy and specificity as high as 0.98. These results suggest that thigmotaxis in the early training stage is a predictive factor for impaired performance in MWM, and machine learning can detect such behavior easily and automatically.

## Introduction

The Morris water maze test (MWM), which was originally invented by Richard G. Morris in 1983, is one of the most popular and established behavioral tests to evaluate rodents’ spatial learning and memory [1–2].

Although this is a useful behavioral test, the results are susceptible to various test conditions. For example, it is reported that the performance in MWM is impaired under stressful situations such as a bright light condition, and the percentage of thigmotaxis increases [3]. Thigmotaxis refers to an animal’s propensity to move along the edge of its environment. This behavior is used as a marker of stress for rodents in open-field situations including MWM tasks [4]. If the subject shows thigmotaxis, the mean escape latency is prolonged, since the subject has difficulty in finding the platform location. As a result, spatial learning ability cannot be appropriately evaluated [5–6]. Therefore, the early detection of thigmotaxis is important for optimal analysis. However, there are few reports discussing the ideal timing of thigmotaxis detection in MWM.

In order to determine the strongest influencing factor, we classified swim path trajectories into six types and assessed which type seen in the early stage of training was associated with impaired performance on the final day. In addition, we made an automatic image recognition model and evaluated the accuracy of swim path trajectory detection.

Previously, there were some attempts for identifying mouse’s swim strategies in MWM. In 2000, Dalm S et al reported that image analysis system could enable the quantification of swim patterns. They used cumulative distance to platform to characterize the mouse’s exploration [7]. Though thigmotaxis is not mentioned in the paper, this method should be also applicable to detect the specific trajectory. However, this parameter can be obtained only with the specific image analysis system, EthoVision 1.7 and cannot be used for detailed classification.

In the same year, Wolfer D et al proposed a novel method to apply principal component analysis (PCA) for MWM to detect the cofounding factor among the determinants of cognitive function [8]. This study should be the epochal one that first employed the machine learning method in MWM analysis.

Later on, Graziano A et al put forward the automatic recognition of explorative strategies with linear discriminant analysis (LDA). They defined four regions of interest (ROI) inside the arena and set twenty-eight dependent variables in order to classify seven different trajectories. They used discriminant function (DF) which is a sort of linear regression and achieved high classification accuracy for each strategy [9]. They did not use the image data itself but the extracted twenty-eight feature quantities which were selected or defined by human researcher. Illouz T et al also used a supervised machine learning method, support vector machine (SVM). In their model, they did not use the pixel data itself but used manually determined eleven features as input data [10].

Although these previous methods are efficient in recognition accuracy, they require expert knowledge for the model construction and sometimes not practical in ordinary laboratory. Therefore, we decided to employ artificial neural network (ANN) model for image recognition in this study. Since ANN is a useful data-driven model, we do not need to manually select the feature quantities. In this study, we just gave raw image data to ANN model and conducted supervised machine learning. Considering its advantage in image recognition, we used convolutional neural network (CNN) to detect swim trajectories among several kinds of ANN architectures.

## Materials and methods

This study was performed in accordance with the National Institutes of Health guidelines for the use of experimental animals. All animal studies were reviewed and approved by the Animal Studies Committee of Ehime University. Minimal dataset required to replicate our study findings are available from the online repository (https://figshare.com/s/90d7b2d038551efe08ec).

### Animals

Fifty male C57B1/6 mice (wild type, WT) which underwent MWM from July 2014 to July 2017 were enrolled in this analysis. Twenty eight mice were treated to produce bilateral common carotid artery stenosis (BCAS) at the age of 10 weeks.

The animals were housed in a room with a 12-hour light/dark cycle with a temperature of 25±1°C. They were given standard laboratory chow (MF; Oriental Yeast Co., Ltd., Tokyo, Japan) and water ad libitum.

### Bilateral common carotid artery stenosis (BCAS)

In order to assess the influence of cerebral ischemia on behavioral pattern, we employed vascular dementia mouse model in addition to the control mouse.

Among all fifty mice enrolled in this study, twenty-eight mice underwent BCAS surgery at 10 weeks old. Micro-coils with an inner diameter of 0.18 mm, pitch 0.5 mm and total length 2.5 mm were used to create artificial stenosis in the bilateral common carotid arteries (CCAs). Before the procedure, mice were anesthetized with sodium pentobarbital (50 mg/kg intraperitoneal). Through a midline cervical incision, both CCAs were exposed and freed from their sheaths. The artery was gently lifted with a silk suture and then placed between the loops of a micro-coil. The micro-coil was twined around by rotating it around the CCA. Then another micro-coil was applied to the other CCA. After placing the coils, the incision was closed with sutures. More detailed procedural information is available in a previous report [11].

### Morris water maze test

**Fig 1:**
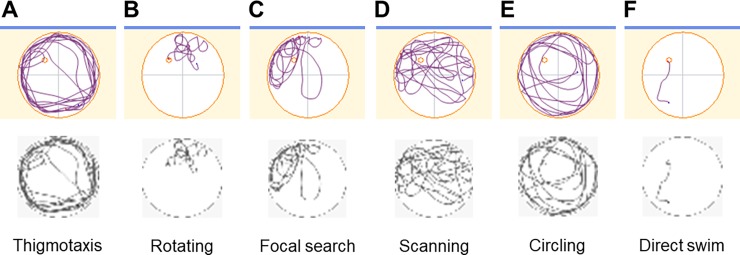
Representative trajectories in swim path classification and converted images. Swim path trajectories were classified into the following six classes: A) Thigmotaxis, B) Rotating, C) Focal search, D) Scanning, E) Circling, and F) Direct swim. Class labeling was conducted by a human researcher before use in the neural network model. Converted grayscale images are shown just below the original images.

MWM was performed at 16 weeks of age as described previously [12]. Mice were trained 5 times a day at 20-min intervals for 5 consecutive days. In each trial, mice were given 120 sec to find the platform. Swimming was video-tracked (AnyMaze; Stoelting Co., Wood Dale, IL), and the mean escape latency was recorded. Swim path trajectories were obtained as image files and manually labelled according to the six classes described in the previous report [13]. Each strategy was defined as follows: Thigmotaxis: Swimming in the outer 10% close to walls. Mouse swims almost exclusively in the periphery; Rotating: Swim with a rotation waking a many small circles or twisting paths. This trajectory reflects the mouse’s trial and error in limited area; Focal search: Swim within two quadrants of the arena. The trajectory is consisted of mainly linear trajectories; Scanning: Swimming consists of wide and repeated foraging around the pool. The trajectories are not circular but jagged with sudden changes in direction and velocity; Circling: Moves away from the wall to explore the pool, usually drawing circular trajectory; Direct swim: swim fast and straight from the starting point to the platform. Animal adjusts its swimming trajectory while approaching the platform. If some traits were mixed within one trial, most prominent trajectory was adopted as a class label. Representative trajectories are shown in Fig 1.

In addition, we scored the cognitive performance of each training trial in reference to a previous study [10] according to the following scale: Thigmotaxis: 0, Scanning: 1, Circling: 2, Focal search: 3, Rotating: 4, Direct swim: 5. Average cognitive score for a day in each mouse was calculated and used for the analysis.

We defined the day 1 to day 2 as ‘early training stage’ and day 4 to day 5 as ‘late training stage’ and assessed the frequency of exploratory strategy in each period. In this method, one mouse could be classified into multiple groups, because mouse swims five times within a day.

### Dataset preparation

For the construction of image recognition model, all swim path images on early training stage (n = 500) were collected. The original format was a Microsoft Windows Bitmap Image (BMP) file with 140x120 pixel size and 32-bit color data. All image data were converted to grayscale pictures of reduced pixel size using a free image processing tool of Python interpreter (Pillow; Alex Clark and Contributors). We tested the model performance for the following three sizes: 72x72, 48x48, and 24x24.

The dataset was divided into two sub-datasets; 80% of the data were used for the training stage and the other 20% were used for validation. Whole image files were randomly rearranged before being assigned to each sub-dataset. Pixel values derived from each image file were divided by 255 for standardization and passed to the following neural network model as input data.

### Convolutional neural network system

**Fig 2:**
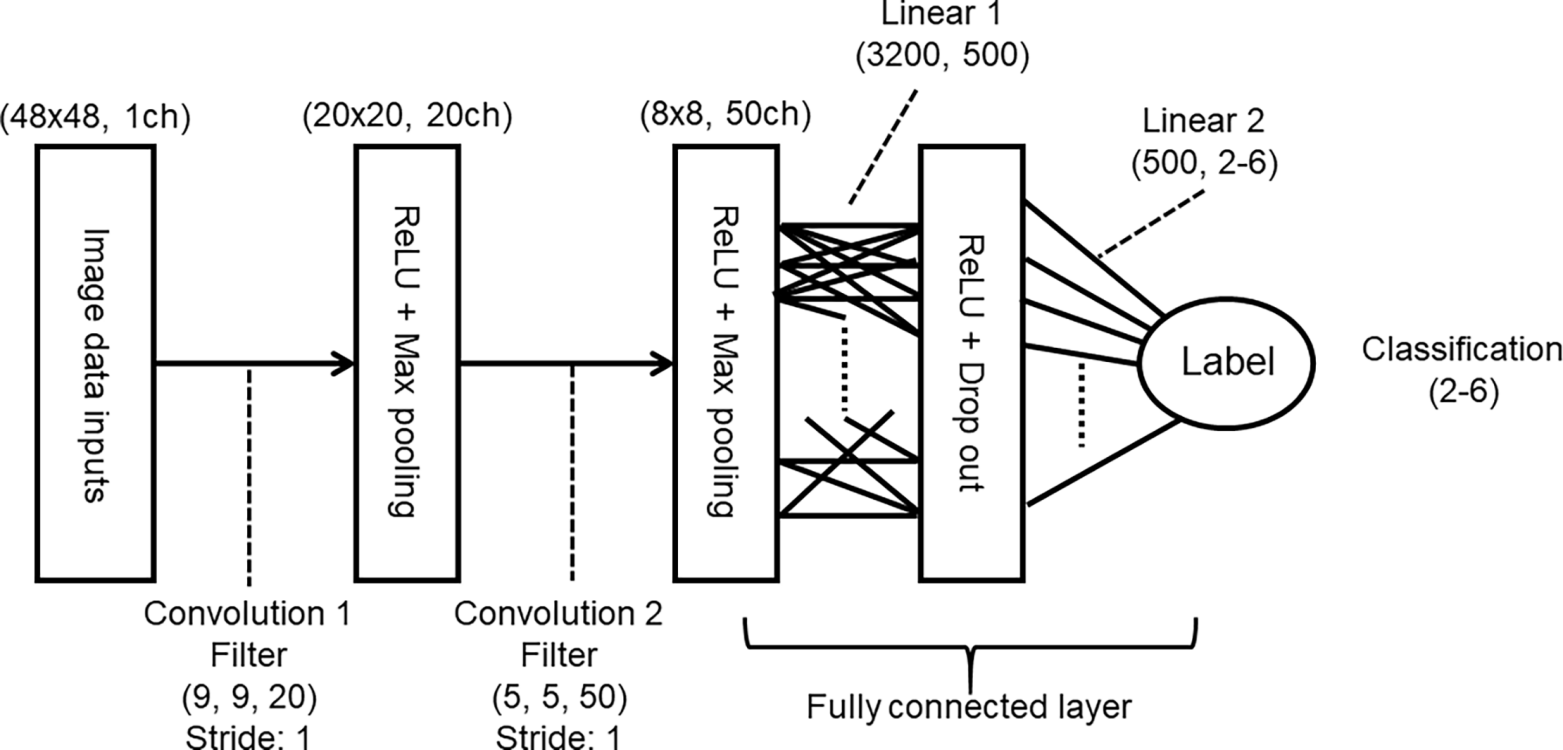
Schema of convolutional neural network model for 48x48. The two left layers are for the convolution process and the two right layers are for linear connections. Values in parentheses are input and output data size in each layer. ReLU: rectified linear unit.

A convolutional neural network (CNN) with two convolution layers and two fully connected layers were used to classify the swim path trajectories in the MWM. The structure of the CNN for 48x48 is shown in Fig 2. At the first convolution layer, 20 kernels with 9x9 pixel size were used. Fifty kernels with 5x5 pixel size were used in the following convolution layer. Down sampling was performed by max pooling with stride of 1 in each process. A rectified linear unit was also used in this process as an activation function. As for 72x72 pictures, the kernel sizes were set to 13x13 and 7x7 so as to fix the convolution ratio. Similarly, 5x5 and 3x3 kernels were used for 24x24 pictures.

The middle layer in the fully connected layer had 500 nodes, and drop out method was used to avoid the overfitting phenomenon. Values were finally passed to the output node as a multidimensional vector according to the number of classes in the classification (2, 3 or 6).

Loss function was defined by the cross entropy method, and adaptive moment estimation (Adam) was selected as the optimization algorithm to minimize the loss function. Necessary gradients were calculated by backward propagation method (backpropagation). After optimization 50 times with the training dataset, we obtained an updated model to use in the validation stage.

The accuracy of the CNN was determined by a cross validation method. As described above, 20% of the whole data were used for validation. Repeated holdout cross-validation was performed 10 times for each randomly rearranged dataset, and the average score was employed as the valid outcome. Sensitivity and specificity were also calculated if applicable.

All these modeling processes were provided by Chainer, an open source framework for deep learning [14]. We used an ordinary laptop computer with CPU of Intel Core i7-3517U 1.9GHz and 4GB DDR3-SDRAM (Dell System XPS L322X, Dell Inc., TX).

### Statistical analysis

All data are presented as mean ± SEM. Data were analyzed with F-test followed by Student’s or Welch’s t-test to assess the difference between two groups. One way ANOVA was used for multiple comparison analysis. A value of P<0.05 was considered statistically significant. SciPy module, the open source scientific tools for Python was used for statistical analysis. Statcel 3 (OMS Inc., Japan), add-in software for Microsoft Excel was also used for supplemental analysis.

## Results

### Swim path characteristics in each stage of training

Thigmotaxis was seen in 8.8% of all trajectories in early training stage (Table 1). In both groups, ‘Scanning’ was most frequently observed in early training stage and the frequency of ‘Direct swim’ became the highest in late training stage (Table 2).

**Table 1:** Mouse characteristics in early training stage.

	Trials (number)	Thigmotaxis (%)	Rotating (%)	Focal search (%)	Scanning (%)	Circling (%)	Direct swim (%)	Escape latency (sec)
All mice	500 (50)	44 (8.8)	85 (17.0)	87 (17.4)	145 (29.0)	84 (18.8)	55 (11.0)	77.1 ± 4.7
Control	220 (22)	15 (6.8)	41 (18.6)	41 (18.6)	68 (30.9)	31 (14.1)	24 (10.9)	70.1 ± 7.7
BCAS	280 (28)	30 (10.7)	44 (15.7)	49 (17.5)	73 (26.1)	54 (19.3)	30 (10.7)	82.5 ± 5.9

There was no significant difference in the frequencies of swim path trajectories. Escape latency refers to the mean time to reach the platform on day 2.

**Table 2:** Mouse characteristics in late training stage.

	Trials (number)	Thigmotaxis (%)	Rotating (%)	Focal search (%)	Scanning (%)	Circling (%)	Direct swim (%)	Escape latency (sec)
All mice	500 (50)	34 (6.8)	109 (21.8)	103 (20.6)	53 (10.6)	48 (9.6)	153 (30.6)	45.3 ± 4.3
Control	220 (22)	10 (4.5)	51 (23.2)	49 (22.3)	18 (8.2)	14 (6.4)	78 (35.5)	35.8 ± 8.2
BCAS	280 (28)	16 (5.7)	57 (20.4)	53 (18.9)	40 (14.3)**	37 (13.2)*	77 (27.5)	52.7 ± 5.5*

The frequency of ‘Scanning’ and ‘Circling’ were significantly higher in BCAS group. Escape latency refers to the mean time to reach the platform on day 5.

*p<0.05

**p<0.01 vs Control.

In early training stage, there was no significant difference in the frequencies of particular trajectories between mouse groups. In late training stage, control mice tended to show more ‘Direct swim’ compared to the BCAS groups (p = 0.06). In contrast, BCAS treated mice showed significantly more ‘Scanning’ and ‘Circling’ than control group and the mean escape latency was significantly prolonged.

There was no significant difference in the average swim speed (m/sec) between control and BCAS group (0.117±0.014 vs 0.112±0.003, p = 0.77).

### Effect of dominating trajectory class on final outcome

In order to determine the predictive factor for the poor performance, we identified the mouse which showed a specific exploratory strategy at least once in the early trials.

**Fig 3:**
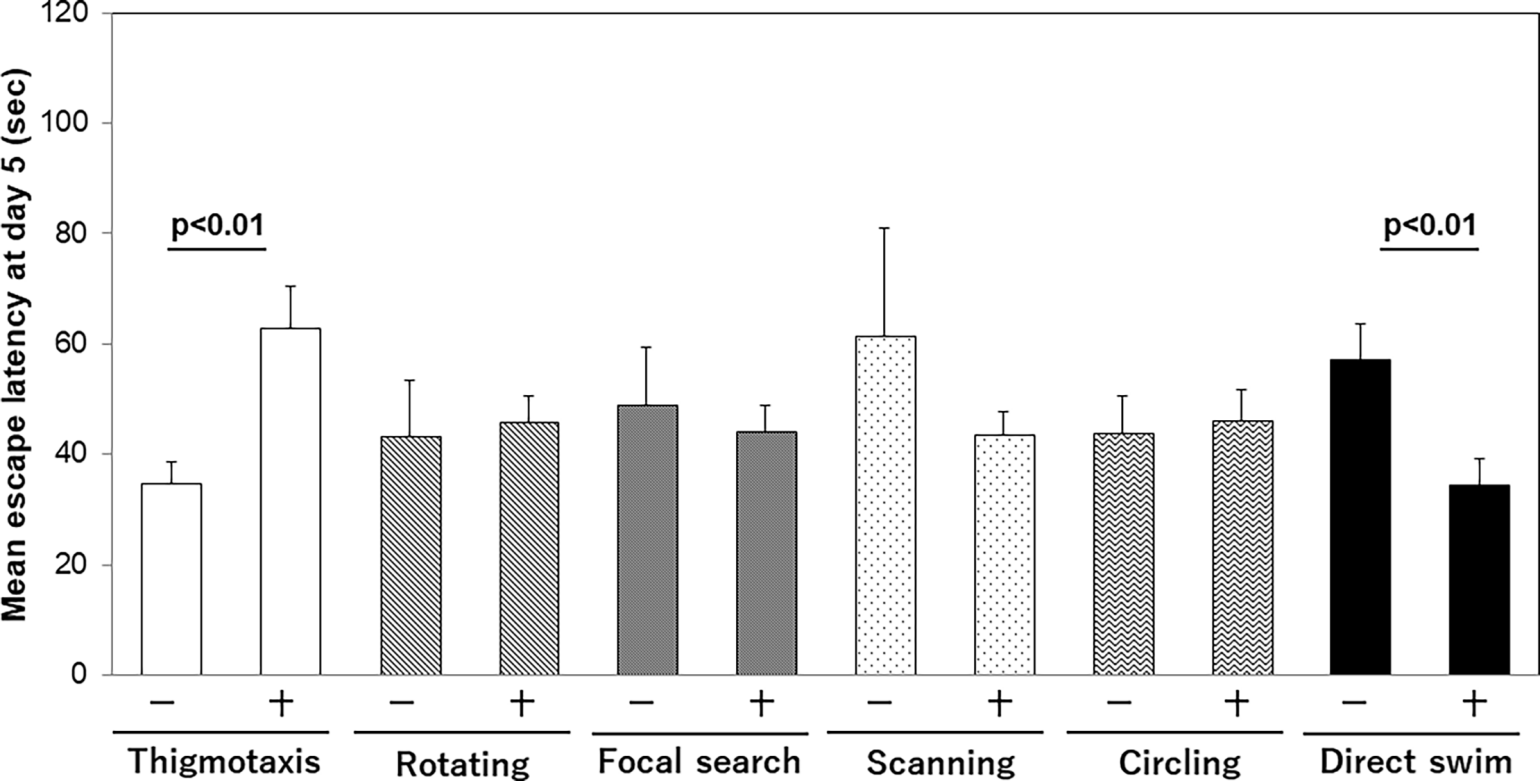
Effect of dominating trajectory on final outcome. Each group was defined as a certain trajectory being seen at least once during the observational days. Presence of certain trajectory is indicated by + symbol. When mouse showed thigmotaxis or direct swim in the early stage, the final outcome was significantly affected.

We compared the mean escape latency on day 5 according to the existence of particular swim path trajectories. Of all the six classes, only thigmotaxis and direct swim affected the final outcome significantly. The group with thigmotaxis in the early training stage showed significantly longer escape latency compared to mice without thigmotaxis (67.8±7.9 vs 38.6±5, p = 0.03). In contrast, the group with direct swim in the early training stage showed significantly shorter escape latency than that in the other groups (38.4±5.2 vs 68.1±8.2, p = 0.002). This trend was also true in the case when the trajectories were only observed on day 1 or day 2 (Fig 3).

### Transition of cognitive scores and its relation to early stage strategy

**Fig 4:**
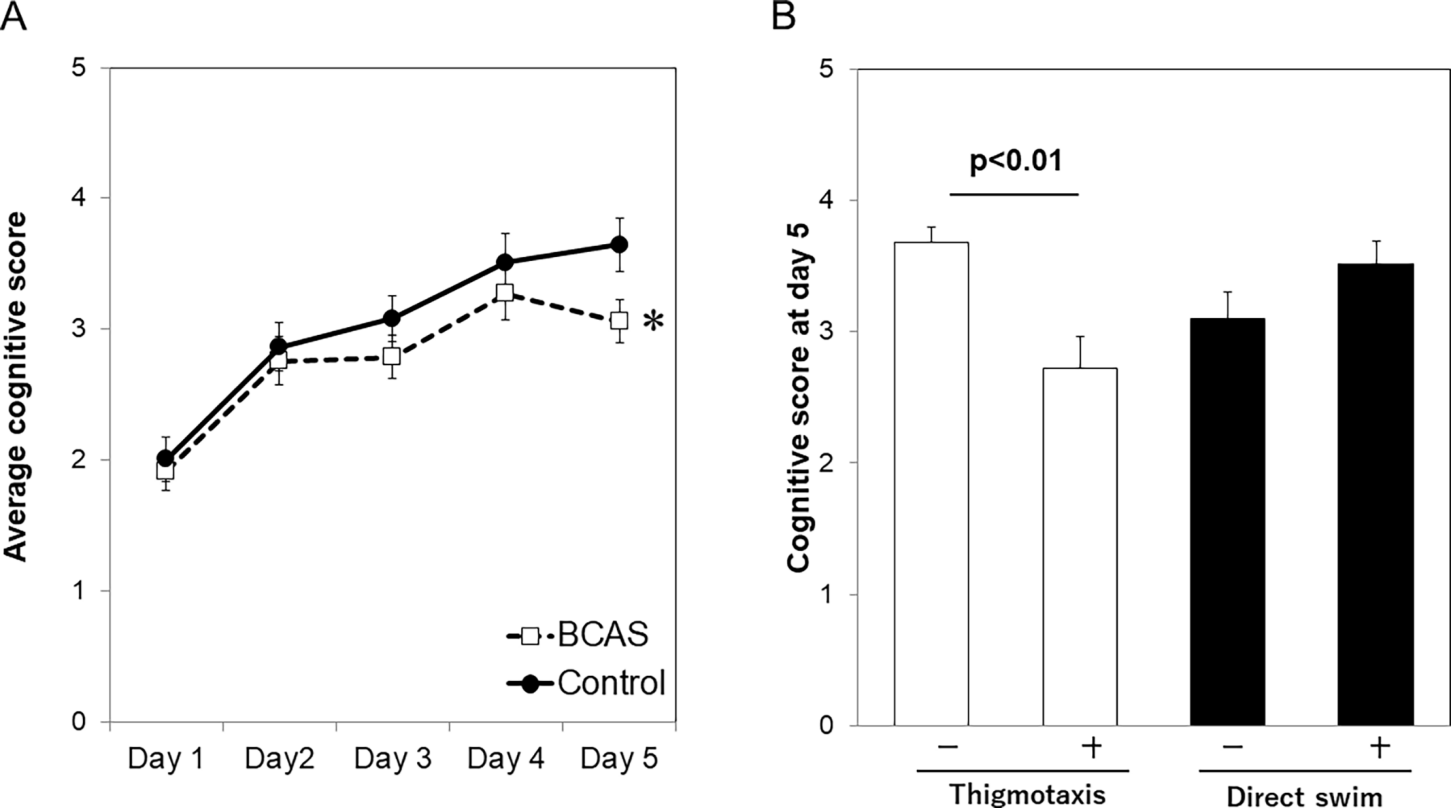
Transition of cognitive scores and its relation to the early stage swim strategy. Average cognitive score increased as the training proceeds in both control and BCAS groups (A). The existence of thigmotaxis in early training stage significantly affected the cognitive score in day 5 (B). *p<0.05 vs control group.

As shown in Fig 4A, average cognitive score increased as the training proceeded in both control and BCAS group. However, the cognitive score at day 5 was significantly lower in BCAS group (3.65±0.20 vs 3.06±0.17, p = 0.03). The cognitive score was significantly lower in the group with thigmotaxis than the other group (2.7±0.2 vs 3.7±0.1, p = 0.001) (Fig 4B).

### Accuracy of convolutional neural network model for image recognition

First, we compared the accuracy of two-label (thigmotaxis and others) classification model (2-class model) among three different picture sizes; 72x72, 48x48 and 24x24. As shown in Table 3, the sensitivity gradually increased as the picture size increased. However, these differences were not statistically significant. From the viewpoint of processing time, we employed 48x48 pictures for the further analysis.

**Table 3:** Model performance according to the picture sizes.

Image size for input	Accuracy (%)	Sensitivity	Specificity	Processing time (sec)
72x72	94.7 ± 9.9	0.76 ± 0.04	0.96 ± 0.00	639.9 ± 9.9
48x48	95.6 ± 0.6	0.72 ± 0.03	0.98 ± 0.00	175.4 ± 0.5**
24x24	93.9 ± 0.8	0.66 ± 0.04	0.97 ± 0.00	30.1 ± 0.4††

Two-class recognition refers to distinguishing mice with thigmotaxis from others. Sensitivity for thigmotaxis detection decreases along with the reduced picture size, but there is no significant difference. Processing time is significantly longer in big image processing.

**p<0.01 vs 72x72

††p<0.01 vs 48x48.

2-class model showed significantly higher recognition accuracy compared to six-group classification. The sensitivity of the two-class model was 0.72 and specificity was 0.98 (Table 4). 6-class model showed significantly lower performance compared to other models.

**Table 4:** Accuracy, sensitivity and specificity of recognition model.

Number of classes in classification	Accuracy (%)	Sensitivity	Specificity	Processing time (sec)
2-class	95.6 ± 0.6	0.72 ± 0.03	0.98 ± 0.00	175.4 ± 0.5
6-class	65.2 ± 1.6**	N/A	N/A	176.6 ± 1.1
3-class	91.7 ± 0.8	N/A	N/A	180.6 ± 1.5

This table shows the details of the efficacy of the CNN models. Two-class recognition refers to distinguishing mice with thigmotaxis from others. For three-class recognition, the direct swim label was added to two-class recognition. Six-class recognition classifies image data into all six classes.

**p<0.01 vs 2-class and 3-class.

### Detailed analysis for misclassification in 6-class model

Among all the misclassification through the validation process in 6-class model, the major classification error occurred between ‘Scanning’ and ‘Circling’. Misclassification of ‘Scanning’ to ‘Circling’ accounted for 17.1% and the opposite was 16.8% as shown in Table 5. ‘Thigmotaxis’ accounted for only 9% of the total errors and the most frequent counterpart was ‘Cirlcing’ (5.8%).

**Table 5:** Misclassification matrix in 6-class model.

Labels	Thigmotaxis	Rotating	Focal search	Scanning	Circling	Direct swim
Thigmotaxis	N/A	0.0	2.6	0.3	5.8	0.3
Rotating	0.3	N/A	1.7	1.4	1.2	2.0
Focal search	0.6	3.2	N/A	4.0	3.5	0.9
Scanning	0.3	1.2	9.2	N/A	17.1	1.4
Circling	5.2	2.6	8.1	16.8	N/A	0.0
Direct swim	0.6	4.9	4.0	0.3	0.6	N/A

Rows indicate the class label and the columns refer to the predicted labels. Percentage to the whole misclassification was shown in each cell. Misclassification between ‘Circling’ and ‘Scanning’ was most frequently observed.

## Discussion

Thigmotaxis is one of the most common traits that rodents show in open field behavioral tests including the water maze test. Thigmotaxis is generally thought to be an indicator of anxiety or fear, and is reported to be associated with an elevated level of corticosteroid [3–4]. When thigmotaxis occurs, rodents can seldom find the platform since the exploration process is disturbed. As a result, the mean escape latency is prolonged. Therefore, the frequency of this behavior is an important factor in assessment of an animal’s spatial learning ability. Some reports state that anxiety or emotional stress can impair spatial learning and memory [15–16]. However, it is not clear whether increased anxiety is induced by impaired cognitive function.

We previously reported that chronic cerebral hypo-perfusion with BCAS impaired the performance in MWM [17]. As the locomotor activity is not impaired with BCAS at 30 days after the treatment [18], the prolonged escape latency in MWM is now attributed to the impairment of hippocampal function [19]. However, the effect of chronic ischemia on the frequency of thigmotaxis is not fully examined before.

In our study, about half of the subjects were vascular dementia model mice, and there was no significant difference in the frequency of thigmotaxis in the early training stage. We also demonstrated that the existence of thigmotaxis in the early training stage was significantly associated with longer escape latency on day 5 of the trial.

Besides the escape latency, we assessed the cognitive scores in each trial. The average score in day 5 was significantly lower in BCAS group than control and this should reflect the cognitive dysfunction induced by chronic cerebral hypoperfusion.

Interestingly, the group with thigmotaxis in early training stage showed significantly low cognitive score in day 5. This result suggests the mouse’s behavioral strategy is affected by the state of anxiety. On the other hand, the existence of direct swim did not significantly affect the final cognitive score. We think the frequency of this trajectory do not necessarily mean mouse’s cognitive functions, because direct swim in early training stage includes the incidental landing.

These results suggest that thigmotaxis could affect the cognitive dysfunction but it could be also a potential factor causing underestimation of spatial learning ability. Therefore, we think it is important to identify thigmotaxis in the early stage of training in MWM.

In this study, we employed an ANN model to detect the swim path trajectories.

ANN is recognized as a useful data-driven empirical model inspired by a biological neural network. Since the invention of the backward propagation technique in 1989, ANN has become a major approach in the field of machine learning [20].

CNN is a technique to improve the accuracy of image recognition of ANN [21]. A set of learnable filters (called kernels) is used to extract the feature quantity from the original image data. With these filters, the input data are converted into smaller pixel size with marked feature quantities. CNN simulates neural connections in the human visual cortex and enables more effective learning compared to a conventional ANN. This method is applied for a variety of tasks such as diagnostic imaging in clinical fields [22].

In this study, we made three models with different levels of classification. As expected, the two-class recognition model showed the highest accuracy among all the models. However, we have to admit that this high level of accuracy was due to its high specificity, and the sensitivity was relatively low. Although we think our model is acceptable for the purpose of screening, some improvement should be required.

To begin with, we converted the original bitmap files into small grayscale pixel images so that we could handle the data with an ordinary laptop computer. So, we assessed the relation between the image resolution and model’s performance. In our study, the image resolution did not affect the recognition accuracy and the sensitivity. Therefore, we suppose the reason of low sensitivity for thigmotaxis is due to the relatively small sample number for the validation data (8.8% of the whole trajectories). This problem can be solved by the accumulation of thigmotaxis images in the future.

We tried to apply the CNN model for the other levels of classification, but failed to obtain high accuracy in 6-class model. Therefore, we assessed the content of misclassifications. Among all the classification errors, ‘Scanning’ and ‘Circling’ were most frequently confused with each other and the proportion was about 35% in total. That is, if we combine these two classes into one class, the accuracy improves to the acceptable level. Since both ‘Scanning’ and ‘Circling’ correspond to low cognitive score, this may be a compromise plan for practical use. Of course, increasing sample number should be a most promising way to improve the model performance. More technically, using ‘binary choice tree’ for distinguishing ‘Circling’ from ‘Scanning’ in combination to the supervised learning is a possible option as proposed in the previous study [10].

Apart from these issues, there is some study limitations. As the initial class labeling was conducted by one person, the decision was susceptible to one’s subject. This could influence the classification accuracy in this study.

In contrast to our model, a recent study proposed a detailed classification of swim paths in MWM [23]. The authors constructed a semi-automated classification method that divides a single swim path into segments and classifies them into eight different types of behavior. This method enables the detection of subtle and novel behavioral differences in rodent groups within a single trial. We think the convolutional neural network could be applied for this detailed classification within single trial in the future.

In summary, our study suggests that a particular swim path trajectory in the early training stage is significantly associated with the final outcome, and this pattern could be automatically detected by a CNN model with high accuracy. We think this study will stimulate discussion on the interpretation of thigmotaxis in the MWM test and promote the application of ANN to various behavioral tests.

## Conclusions

A convolutional neural network could recognize thigmotaxis from swim path images in the early training stage, and this was associated with the final outcome in MWM.

## Abbreviations

MWM: Morris water maze
BCAS: Bilateral common carotid artery stenosis
ANN: Artificial neural network
CNN: Convolutional neural network

## References

[1] Morris RG. Spatial localization does not require the presence of local cues. Learn Motiv. 1981;12,239–260

[2] D'Hooge R and De Deyn PP. Applications of the Morris water maze in the study of learning and memory. Brain Res Brain Res Rev. 2001;36,60–90 11516773 10.1016/s0165-0173(01)00067-4

[3] Treit D, Fundytus M. Thigmotaxis as a test for anxiolytic activity in rats. Pharmacol Biochem Behav. 1988 12;31(4):959–62. 3252289 10.1016/0091-3057(88)90413-3

[4] Huang Y, Zhou W, Zhang Y. Bright lighting conditions during testing increase thigmotaxis and impair water maze performance in BALB/c mice. Behav Brain Res. 2012 1 1;226(1):26–31. doi: 10.1016/j.bbr.2011.08.043 21907245 10.1016/j.bbr.2011.08.043

[5] Devan BD, McDonald RJ, White NM. Effects of medial and lateral caudate-putamen lesions on place- and cue-guided behaviors in the water maze: relation to thigmotaxis. Behav Brain Res. 1999 4;100(1–2):5–14. 10212049 10.1016/s0166-4328(98)00107-7

[6] Acheson SK, Moore NL, Kuhn CM, Wilson WA, Swartzwelder HS. The synthetic cannabinoid WIN 55212–2 differentially modulates thigmotaxis but not spatial learning in adolescent and adult animals. Neurosci Lett. 2011 1 10;487(3):411–4. doi: 10.1016/j.neulet.2010.10.067 21055447 10.1016/j.neulet.2010.10.067 PMC3035942

[7] Dalm S., Grootendorst J., De Kloet E.R. and Oitzl M.S., 2000 Quantification of swim patterns in the Morris water maze. Behavior Research Methods, Instruments, & Computers, 32(1), pp.134–139. 10.3758/bf03200795 10758671

[8] Wolfer D.P. and Lipp H.P., 2000 Dissecting the behaviour of transgenic mice: is it the mutation, the genetic background, or the environment?. Experimental physiology, 85(6), pp.627–634. 11187958

[9] Graziano A., Petrosini L. and Bartoletti A., 2003 Automatic recognition of explorative strategies in the Morris water maze. Journal of neuroscience methods, 130(1), pp.33–44. 14583402 10.1016/s0165-0270(03)00187-0

[10] Illouz T., Madar R., Louzon Y., Griffioen K.J. and Okun E., 2016 Unraveling cognitive traits using the Morris water maze unbiased strategy classification (MUST-C) algorithm. Brain, behavior, and immunity, 52, pp.132–144 doi: 10.1016/j.bbi.2015.10.013 26522398 10.1016/j.bbi.2015.10.013

[11] Shibata M, Ohtani R, Ihara M, Tomimoto H. White matter lesions and glial activation in a novel mouse model of chronic cerebral hypoperfusion. Stroke. 2004 11;35(11):2598–603. doi: 10.1161/01.STR.0000143725.19053.60 15472111 10.1161/01.STR.0000143725.19053.60

[12] Sakata A, Mogi M, Iwanami J, Tsukuda K, Min LJ, Horiuchi M et al Sex-different effect of angiotensin II type 2 receptor on ischemic brain injury and cognitive function. Brain Res. 2009 12 1;1300:14–23. doi: 10.1016/j.brainres.2009.08.068 19729000 10.1016/j.brainres.2009.08.068

[13] Tuma J, Kolinko Y, Vozeh F, Cendelin J. Mutation-related differences in exploratory, spatial, and depressive-like behavior in pcd and Lurcher cerebellar mutant mice. Front Behav Neurosci. 2015 5 12;9:116 doi: 10.3389/fnbeh.2015.00116 26029065 10.3389/fnbeh.2015.00116 PMC4429248

[14] Tokui, S, Oono, K, Hido, S, Clayton J. Chainer: a next-generation open source framework for deep learning. Available from: http://learningsys.org/papers/LearningSys_2015_paper_33.pdf

[15] Goodman J, McIntyre CK. Impaired spatial memory and enhanced habit memory in a rat model of post-traumatic stress disorder. Front Pharmacol. 2017 9 22;8:663 doi: 10.3389/fphar.2017.00663 29018340 10.3389/fphar.2017.00663 PMC5614977

[16] Packard MG, Wingard JC. Amygdala and "emotional" modulation of the relative use of multiple memory systems. Neurobiol Learn Mem. 2004 11;82(3):243–52. doi: 10.1016/j.nlm.2004.06.008 15464407 10.1016/j.nlm.2004.06.008

[17] Higaki A, Mogi M, Iwanami J, Min LJ, Bai HY, Shan BS, Kukida M, Kan-No H, Ikeda S, Higaki J, Horiuchi M. Predicting outcome of Morris water maze test in vascular dementia mouse model with deep learning. PLoS One. 2018 2 7;13(2):e0191708 doi: 10.1371/journal.pone.0191708 29415035 10.1371/journal.pone.0191708 PMC5802845

[18] Shibata M, Yamasaki N, Miyakawa T, Kalaria RN, Fujita Y, Ohtani R, Ihara M, Takahashi R, Tomimoto H. Selective impairment of working memory in a mouse model of chronic cerebral hypoperfusion. Stroke. 2007 10;38(10):2826–32. doi: 10.1161/STROKEAHA.107.490151 17761909 10.1161/STROKEAHA.107.490151

[19] Nishio K, Ihara M, Yamasaki N, Kalaria RN, Maki T, Fujita Y, Ito H, Oishi N, Fukuyama H, Miyakawa T, Takahashi R, Tomimoto H. A mouse model characterizing features of vascular dementia with hippocampal atrophy. Stroke. 2010 6;41(6):1278–84. doi: 10.1161/STROKEAHA.110.581686 20448204 10.1161/STROKEAHA.110.581686

[20] Rumelhart DE, McClelland JL, Williams RJ. Learning representations by backpropagation. Nature (323) (1986), pp. 533–5363960136

[21] LeCun Y, Boser B, Denker JS, Henderson D, Howard RE, Hubbard W, Jackel LD. Backpropagation applied to handwritten zip code recognition. Neural Comput. 1 (4): 541–551. 1989

[22] Lakhani P, Sundaram B. Deep learning at chest radiography: automated classification of pulmonary tuberculosis by using convolutional neural networks. Radiology. 2017 8;284(2):574–582. doi: 10.1148/radiol.2017162326 28436741 10.1148/radiol.2017162326

[23] Gehring TV, Luksys G, Sandi C, Vasilaki E. Detailed classification of swimming paths in the Morris Water Maze: multiple strategies within one trial. Sci Rep. 2015 10 1;5:14562 doi: 10.1038/srep14562 26423140 10.1038/srep14562 PMC4589698

